# Urinary tract infection: is it time for a new approach considering a gender perspective and new microbial advances?

**DOI:** 10.3389/fruro.2024.1487858

**Published:** 2024-10-30

**Authors:** María José González, Luciana Robino, Pablo Zunino, Paola Scavone

**Affiliations:** ^1^ Laboratorio de Biofilms Microbianos, Departamento de Microbiología, Instituto de Investigaciones Biológicas Clemente Estable (IIBCE), Montevideo, Uruguay; ^2^ Unidad Academica de Bacteriología y Virología, Facultad de Medicina, Universidad de la República, Montevideo, Uruguay; ^3^ Departamento de Microbiología, Instituto de Investigaciones Biológicas Clemente Estable (IIBCE), Montevideo, Uruguay

**Keywords:** urinary tract infection, definition, diagnosis, urobiome, women

## Abstract

Urinary tract infections (UTIs) are among the most common bacterial infections in humans, particularly affecting women, with significant clinical and socioeconomic impacts. Despite advances in medical research, the diagnostic criteria for UTI have remained practically unchanged since Kass’s seminal work, emphasizing the need for a reevaluation in light of new scientific insights. Recent studies have highlighted the importance of the urobiome, a previously underappreciated community of microorganisms within the urinary tract (UT), and its role in maintaining urogenital health. The gut-bladder axis has emerged as a critical pathway in understanding UTI as a dysbiosis, where imbalances in the microbial community and its relation with the host contribute to infection susceptibility. This review explores the evolving definitions and diagnostic challenges of UTI, particularly in women, and examines the implications of recent discoveries on the urobiome and the gut-bladder axis. Additionally, we discuss the potential of novel therapeutic strategies to restore microbial balance, offering a promising avenue for the therapeutic management of UTIs.

## Magnitude of UTI

Urinary tract infections (UTI), traditionally defined as conditions in which bacteria invade and grow in the UT, whether in the bladder, prostate, ureters, or kidneys (1), are the most common and frequent infections, mainly affecting young women. Approximately 50% of all women and 12% of men and children (2–4) will have an episode of UTI in their lifetime; hence, the magnitude of these types of infections is high. Of the 50% of women who will have a UTI, 20 to 30% will have a recurrence within 3 to 4 months of the first infection (3) despite having received antibiotic treatment. The recurrence of the infection has a significant impact on the quality of life, psychologically affecting people who present symptoms of anxiety and depression (5).

From 1990 to 2019, a global increase in UTIs has been reported. More than 404.6 million people had a UTI, and about 237 thousand people died from this type of infection in that period (6). Recent reports show that the incidence of UTI is increasing. In this context, Latin America has the highest age-standardized incidence rate and highest mortality (6).

The costs associated with UTI include diagnosis, treatment, and loss of workdays, among others, and are around 6 billion dollars annually for the United States alone (7).

## Classification of UTI

UTIs have been for long classified according to the accompanying symptoms (asymptomatic and symptomatic), the site of infection (cystitis or lower UTI and pyelonephritis or upper UTI), or related to the existence of complications (complicated and uncomplicated). Complicated UTIs have been usually described as those individuals who have factors that make effective eradication of uropathogens difficult (8). Such factors may include structural or functional abnormalities of the urogenital tract, prolonged antibiotic therapy, UTI caused by more than one pathogen or multidrug-resistant pathogens, as well as catheterized or immunocompromised people (9, 10).

However, different authors claim that definitions of complicated UTI are not uniform since there is a lack of consensus on standards for different aspects of UTI. For example, guidelines for the proposal of new treatment strategies for uncomplicated and complicated UTIs, and different symptom criteria are not uniform (11). These authors recommend making a distinction, instead, between UTIs with and without systemic involvement.

The classic definition of UTI established that infection occurs when microorganisms reach the UT from the gut and activate an inflammatory response, causing damage and characteristic symptoms (5, 12). The most common symptoms of cystitis (lower UTI), experienced especially by women, are pain and burning when urinating, pain in the lower abdomen, an intense desire to urinate constantly, and cloudy or bad-smelling urine. If the infection reaches the kidneys (pyelonephritis), symptoms include fever, back pain, nausea, and vomiting.

Another relevant aspect of UTI is the high recurrence of the infection. Back in 1994, Russo and colleagues published that the same person could experience episodes of UT infections caused by the same strain but that it was not due to the persistence of the pathogen (13). There, the authors stated that ¨the strain was continually reintroduced from a reservoir in the individual’s environment¨, probably anticipating the introduction of the intracellular bacterial communities (IBC) concept. Thirty years later, we can confirm that Russo’s observations are undoubtedly associated with the formation of IBC, protecting microorganisms from the action of antimicrobials and the immune system. Also, the adaptative immune response is necessary to avoid reinfection with the same microorganism. Is it the immune response failing or should other aspects be considered?

Different authors explain the limitation of triggering an effective adaptative immune response in the UT against uropathogens by the need to control an excessive inflammatory response that could damage the uroepithelium (14). However, several selected bacterial antigens could effectively prevent UTI using an ascending model of infection in mice. In these cases, a significant adaptative immune response was observed when specific markers were measured (15, 16).

Most studies and reviews on this subject omitted nobel knowledge about the complexity of bacteria interaction with the bladder epithelium in UTI. As it was mentioned before, intracellular bacteria are protected from the immune response. Moreover, if we think that there is a resident microbiota in the bladder, it should be reasonable not to mount a response against these microorganisms as happens with other microbiota (i.e. in the gut).

## Women and UTI

Women are the most affected by UTIs: by the age of 32, 50% will have experienced at least one episode, and 20-30% will have a second episode within six months (3). These data do not include asymptomatic bacteriuria (bacteria in the urine but without symptoms), which occurs in 2% of school-age girls, 10% of pregnant women, and 1-5% of elderly women (17).

UTIs peak in women between 18 and 39, coinciding with maximum sexual activity. In men, symptoms are mainly due to benign prostatic hyperplasia. Anatomical differences, such as a shorter urethra and closer proximity to the anus in women, are often cited for the higher prevalence of UTIs in women, though no experimental evidence supports this (18). Male infants and elderly men have similar or higher UTI rates compared to females (19), suggesting urethral length alone does not explain the gender difference (20, 21). Other factors like hormonal changes, pregnancy, and urological conditions should also be considered (22).

As mentioned above, while women suffer from UTI from school age, in men, it is more common after 60 years of age. Taking into account that it mainly affects women, directly impacting their quality of life, these infections increase the existing gender gap in the workplace and all aspects of their lives. As they are generally recurrent infections with low death risk, UTIs tend to become naturalized and undervalued. Patients who suffer from them are generally not given the day off from work, and the appearance of symptoms can lead to absenteeism from work, mental health problems associated with having recurrent infections (such as depression and anxiety), and, in many cases, not being able to work (5). Leading an everyday life, such as going to the bathroom calmly, exercising, and even walking normally, could become a nightmare.

Adding to this situation is the issue of overdiagnosis, or the use of empirical treatment to anticipate an accurate diagnosis. While the diagnosis of a UTI is made and the causative pathogen along with its antibiotic resistance profile is identified (with results obtained within 2 to 3 days), patients are treated empirically. Currently, the clinical guidelines that define UTIs are open to ambiguous interpretations, often leading to the unnecessary administration of broad-spectrum antibiotics (23). Surveillance data accumulated by The Global Prevalence of Infections in Urology (GPIU) suggests that approximately 56% of patients in urology units were prescribed antibiotics, of which 26% were for confirmed UTIs, while 21% were for suspected UTIs (24, 25).

## Diagnosis

The diagnosis of UTI is still based on suggestive clinical symptoms and confirmation by urine culture and the development of bacteria with a significant count. Urine culture remains the gold standard for diagnosis, providing data on the etiological agent and its antibiotic susceptibility. Consultation due to urinary symptoms is a frequent reason for attendance at health centers. Still, in a non-negligible percentage of these cases, bacteria are not detected through conventional urine culture.

Other researchers demonstrated that 24% of patients who consulted for typical lower UTI symptoms and presented pyuria in the urine test did not present bacterial growth in conventional urine culture (26). Despite being the gold standard, not all microorganisms are recovered with the classic urine culture method.

Different authors have proposed that uropathogenic bacterial strains (e.g. *E. coli*) may not be eliminated after antibiotic-based treatments, entering a temporarily dormant state, non-detectable using standard culture methods. It must be considered that bacteria in the viable but nonculturable (VBNC) state fail to multiply on artificial culture media that would support their growth under routine conditions (27). Therefore, recurrent UTI can be caused by bacteria in this state. Studies by Anderson et al. have reported that VBNC cells were present not only in mice but also in human urine (28).

In recent years, new insights into the pathogenesis of UTIs have shed light on the difficulties of diagnosing and treating UTIs. For example, the presence of *E. coli* IBC in the bladder epithelium has been demonstrated. *E. coli* can invade the superficial urothelial cells in early infection stages and rapidly multiply in their interior, forming multicellular structures similar to biofilms (29). Therefore, *E. coli* could escape from the immune response and resist treatments based on antibiotics with low intracellular penetration (30).

Also, uropathogenic *Escherichia coli* (UPEC) can form quiescent intracellular reservoirs (QIR), more persistent aggrupations of intracellular cells than IBC, formed within the endosomes of cells located at the deep layers of the uroepithelium (31).

Other bacterial species have also been reported as capable of forming IBC, like *P. mirabilis*, *K. pneumoniae* and *P. aeruginosa* (32–35). Moreover, intracellular bacterial communities of *Stenotrophomonas maltophili*a, *Staphylococcus* spp., and *Enterobacter cloacae* have been recently described in human uroepithelial cells (35).

Bacteria forming these multicellular structures can be protected against the action of antibiotics and the immune system, and not be detected as freely swimming bacteria in the urine of patients with UTI when using standard diagnostic methods.

For the interpretation of the urine culture, the criterion described by Kass in 1957 consists of counting colony-forming units (CFU) per milliliter of fresh urine sown (36). The presence of 100,000 or more CFU per milliliter of urine is considered a predictor of the presence of the infection. Although these cutoff points were established more than 75 years ago, they are still in use today. However, this is not absolute since even in patients with characteristic symptoms of UTI, and with pathological urine examination due to the presence of leukocytes, bacterial growth is often not detected in urine cultures.

In the post-genomic era, we are still cultivating 1-10 ul of urine to diagnose UTI. It is mandatory to include more accurate diagnosis systems that could range from using the Expanded Quantitative Urine Culture (EQUC) to evaluating IBC in desquamated cells or urobiome analysis. Moreover, interesting results have been obtained based on DNA sequencing. McDonald et al. reported that in a phase II study, 44 patients showed positive results in DNA sequencing, while only 13 had positive urine culture tests. In the case of patients with a negative culture, they received treatment according to the generated NGS data, improving their symptoms (37).

On the other hand, it is also important to consider that recovery of more than 1 x 10^5^ CFU/ml does not always denote UTI requiring a subsequent antibiotic prescription. For example, asymptomatic bacteriuria (ASB) implies the presence of one or more bacterial species growing in the urine without signs or symptoms attributable to UTI (38). In most of these cases, antibiotic treatment is not prescribed. Moreover, different authors have proposed that *E. coli* recovered from people with ASB can exert beneficial effects when used as live biotherapeutic products (39).

## Urobiome

In recent years, science has overturned one of the great paradigms that were in force in medicine until recently: the sterility of the UT, and, therefore, of urine (40). This paradigm changed when the urinary commensal microbiota was identified for the first time around 2010, with *Lactobacillus* and *Streptococcus* being the most common genera (41, 42).

The increase in the use of high-throughput approaches has demonstrated that the urinary microbiome is more complex than previously believed (43). The urobiome composition includes not only bacteria but also viruses and fungi, although the bacterial component has been the most extensively studied. The virome, and its role in interaction with the microbiome in both health and disease, still requires further investigation (44). Several viruses, such as human papillomavirus (HPV), polyomavirus, and cytomegalovirus (HCMV), have been reported in the bladder. These viruses are implicated in chronic inflammation, which can lead to cancer development. Bacteriophages, a part of the virome, also represent a promising biotechnological strategy for treating urinary tract infections (UTIs) by targeting specific bacterial pathogens. In contrast, less is known about the role of fungi, and their interactions within the urobiome are less characterized. Some of the pathogenic fungi reported in the urobiome include *Candida albicans*, *C. glabrata*, *Cryptococcus neoformans*, and *Aspergillus fumigatus*, among others (44).

Like other human microbiomes, there is a considerable variation among individual’s urinary microbial diversity and composition. There are important factors that strongly influence variation among individuals, like gender and age (45). Also, controversy exists among different authors on the influence of sample size, collection method, and the technique used to generate valid and comparable results about urobiome composition, diversity, and abundance (46).

Due to the aforementioned, it is necessary to understand how the urinary microbiota, now usually called urobiome, has a relevant role in maintaining balance and how it may interact with the various pathogens that cause urinary UTI.

Although the composition of the urine microbiota related to diverse diseases is under debate, it is assumed that the increase of Enterobacterales and a depletion of *lactobacilli* can be associated with the risk of developing a UTI (46).

Although the origin of the urinary microbiome has not been fully understood so far, the gut and vaginal microbiomes, as well as the external environment, have been proposed as the primary colonization sources (47).

The urinary microbiome displays a wide range of beneficial functions, like the control of pathogenic microorganisms, modulation of the immune response, and promotion of a healthy mucosal barrier, while it influences the development of diverse bladder diseases like bladder cancer, benign prostatic hyperplasia, urgency urinary incontinence, overactive bladder syndrome, interstitial cystitis, bladder pain syndrome, and UTIs (43, 48).

Several researchers are even beginning to talk about the existence of a bidirectional bladder-gut-brain axis, where stress, lack of social interaction, infections or intestinal syndromes, and even emotional relationships can directly influence the development of urogenital disorders (49).

The role of the urinary microbiome is critical in the onset of diseases related to the urinary tract. A decrease in the urobiome diversity is often related to patients suffering from overactive bladder, particularly those with severe symptomatology (50). Moreover, patients suffering from bladder diseases associated with depression or anxiety showed significant changes in the urobiome structure compared with patients who did not suffer from those conditions.

Several authors have proposed relations among the gut and bladder microbiomes. The urinary microbiome composition may be strongly influenced by the gut microbiota, among other factors. It is now assumed that gut disorders may be associated with mental health conditions like anxiety, depression, or stress. Under these circumstances, the communication between the bladder and the brain-gut axis might affect permeability, inflammation, as well as infectious etiology and dysbiosis in bladder diseases (51).

Until now, we have been able to observe that in addition to differences in the abundance of various microorganisms in healthy individuals and those with the infection, there are also differences between the microbiotas of women and men.

In addition, it was found that at least half of people with UTIs have intracellular bacteria, which means that within the cells of the bladder, bacteria “hide” and survive treatments (35).

Understanding that patients must be treated with a comprehensive perspective that addresses them as a whole, working in the search for a better and faster diagnosis and complementary and alternative treatments to antibiotics can contribute to improving the quality of life, mainly of women who suffer from these infections recurrently.

## UTI as a dysbiosis: therapeutic implications

The traditional definition of UTI should be revised since a commensal urinary microbiota has been discovered and recognized. The presence and multiplication of microorganisms could no longer per se define a pathological condition if a commensal microbiological community permanently inhabits the UT. Potentially pathogenic bacteria interact with native microorganisms in the genitourinary tract in a dynamic and balanced situation. When homeostasis of this system is affected, dysbiosis occurs, and certain bacterial strains become predominant. Usually, these strains have particular virulence or fitness attributes that favor overcoming the defense mechanisms of the UT, including the neutralizing effect of the native microbiome (52).

UTI are generally treated with antibiotics, being the second cause of antibiotic prescription after otitis media (53). These treatments are generally successful in infections involving the lower tract that course without systemic repercussions.

For cystitis, the first-line antibiotic treatments are fosfomycin, trometamol and nitrofurantoin. B-lactams like first and second-generation cephalosporins and trimethoprim-sulfamethoxazole are alternative options (54).

In the cases of uncomplicated pyelonephritis, second and third-generation cephalosporins are recommended as the first-line options. Fluoroquinolones are another alternative, but high resistance in some regions and adverse effects at the neuromuscular, bone and musculoskeletal levels limit their use (55).

However, antibiotic effectiveness decreases in recurrent UTI, in catheterized patients or those with anatomical abnormalities in the UTt or concomitant diseases like diabetes or when there are stones that act as niches for bacterial persistence (56).

Moreover, antibiotics may contribute to urinary microbiome dysbiosis since therapies based on antibiotic prescriptions severely affect commensal microbiota (57). For example, it has been found that the use of antibiotics in UTI treatments depletes resident microorganisms in the UT, like lactobacilli (58, 59).

Moreover, uropathogens like UPEC may not always be eradicated after treatments with antibiotics, due to intracellular bacterial persistence. In these cases, symptoms may be temporarily alleviated since the bacterial load is diminished, but the occurrence of recurrent UTI is still latent (39).

Little progress has been made in the last decades in innovative and effective diagnoses and therapies for UTI. Therefore, alternatives to traditional antibiotic-based therapy are crucially needed to arrive at effective treatments for recurrent UTI, overcome the problem of antimicrobial resistance, and avoid possible side effects in patients (60).

The emergence of antibiotic resistance has been recognized as a severe problem of Public Health, and uropathogenic bacteria, in particular, have shown a constant increase in resistance to different antimicrobials in the last two decades (61).

For these reasons, effective prevention and treatment of UTIs constitute an enormous medical challenge that must be constantly updated. From this perspective, the native urinary microbiome appears to be a pivotal actor in the management of UTIs.

Different authors have observed important differences in microbiota, even between healthy people. This situation makes defining a healthy microbiome difficult (61). Therefore, it will be important to consider patients’ particularities in the near future when considering therapeutic measures to prevent or treat UTI.

Different strategies are being proposed to improve the therapeutic management of UTI, including antimicrobial stewardship, diagnostic improvement, different kinds of supplements (e.g., cranberry extract, D-mannose, methenamine hippurate), improvement in antimicrobials delivery (e.g., nanoparticles), antimicrobial peptides, bacteriophages, vaccines, hormones, and microbiome modulation (e.g. prebiotics, probiotics, postbiotics, fecal transplant) (62, 63).

This comprehensive approach to understanding UTIs and the human urobiome encourages the design of alternative therapies to reduce symptoms and prevent pathogen proliferation and infection recurrence. The healthy urobiome could be a valuable source of microorganisms to restore the bladder environment and reduce symptoms, mainly in women (64).

## Conclusions

Knowledge about the UT has rapidly evolved in recent years. This novel information can give researchers and medical staff new tools to improve the diagnosis of UTI and propose new strategies to prevent or treat these infections.

Current diagnostic and therapeutic measures have remained almost unchanged along decades, so a deep revision using this novel information is needed. Specific knowledge about women and UTI susceptibility and recurrence must be updated and taken into account.

The urinary microbiome appears as a central actor that must be considered to face this challenge.
